# Fever of Unknown Origin: Could It Be a Pheochromocytoma? A Case Report and Review of the Literature

**DOI:** 10.1155/2018/3792691

**Published:** 2018-07-03

**Authors:** Uzma Mohammad Siddiqui, Stephany Matta, Mireya A. Wessolossky, Richard Haas

**Affiliations:** ^1^Department of Endocrinology, Diabetes & Metabolism, University of Massachusetts Medical School, 55 Lake Avenue North, Worcester, MA 01655, USA; ^2^Department of Internal Medicine, University of Massachusetts Medical School, 55 Lake Avenue North, Worcester, MA 01655, USA; ^3^Department of Infectious Disease, University of Massachusetts Medical School, 55 Lake Avenue North, Worcester, MA 01655, USA

## Abstract

Pheochromocytomas are rare tumors that arise from the adrenal medulla, with an incidence of less than 1 per 100,000 person-years. These tumors are characterized by excess catecholamine secretion and classically present with the triad of headaches, palpitations, and sweating episodes. However, the clinical presentation can be quite variable. Herein, we present a patient who presented with persistent fevers. An adrenal mass was incidentally discovered during the extensive investigation for the fever of unknown origin. Consequently, blood and urine tests were done and found to be consistent with a pheochromocytoma. The resection of this pheochromocytoma resulted in resolution of fevers. It is hypothesized that fevers in patients with pheochromocytomas occur due to the excess catecholamine or possibly due to interleukins. This clinical presentation serves as a learning point that adrenal incidentalomas in the setting of fever of unknown origin should not be ignored. It also reminds clinicians that pheochromocytomas which present with fevers may have tumor necrosis and many such patients are at risk for multisystem crises.

## 1. Introduction

The first report of a pheochromocytoma emerged in the late 19th century [1]. Pheochromocytomas are rare catecholamine secreting tumors with an estimated incidence of 0.5 to 0.8 per 100,000 person-years in the US [2, 3]. Reports from autopsy studies have revealed that many pheochromocytomas remain undetected till death [1]. These tumors are generally diagnosed either incidentally, due to symptoms, or as part of screening in familial syndromes. Although they may be diagnosed at any age, most sporadic cases are diagnosed after the 4th decade in life [3]. These tumors arise in the chromaffin cells of the adrenal medulla, although they could arise extra-adrenally as well. Some of the most commonly reported symptoms include headaches, sweating, and palpitations. Up to 98% of patients with pheochromocytomas also have hypertension; half of whom may be only intermittently hypertensive [4]. It has been repeatedly reported that pheochromocytomas have a very heterogenous presentation. Up to two-thirds of all patients with pheochromocytomas could also have a fever [4]. However, pheochromocytomas are rarely diagnosed based primarily on fevers [5–7].

A fever of unknown origin (FUO) in a patient is described as temperature higher than 38.3 C (100.9 F) persisting for more than three weeks with no obvious source despite extensive workup. The most prevalent causes of FUOs include infections, connective tissue diseases, and malignancies.

Herein, we describe a patient who presented as a FUO, and the cause was found to be a pheochromocytoma.

## 2. Case Presentation

A 64-year-old woman was referred to the infectious diseases clinic in January 2016. She reported that, for the prior 1 month, she had had daily fevers up to 102 degrees Fahrenheit associated with night sweats. She was also suffering from extreme muscle aches, dry cough, headache, fatigue, and weight loss of 8 pounds. Other medical history includes hypertension and anxiety. She is married and has worked as a teacher's assistant in a kindergarten for the past 30 years. She denied smoking or consuming alcohol. She denied recent travel and reported she had no pets. Her parents had cardiovascular disease and her twin sister had breast cancer.

Lab investigations revealed a white count of 14 th/mm3 (4.3-10.3) with a hematocrit of 33.5% (37-47). Absolute neutrophil count was 10 th/mm3 (1.6-7.5). ESR was >120 mm/hr (0-15) and CRP was 238 mg/L (<10). Thyroid stimulating hormone was 1.19 uIU/mL (0.28-3.89). Serum and urine electrophoresis demonstrated no evidence of a monoclonal spike. Blood cultures and urine cultures were collected and negative. Tests for Lyme Antibody screen, Bartonella IgG/IgM, Anaplasma polymerase chain reaction (PCR), and Babesia PCR screen were negative.

As part of the workup the patient underwent CAT scans of the abdomen and chest in February 2016. This revealed a 3.8 x 2.9 x 5 cm heterogenous enhancing lesion in the left adrenal gland. The right adrenal gland was seen to be normal. A random urine normetanephrine was seen to be 2917 mcg/gram of creatinine (108-524). The patient was then referred to our endocrinology clinic for further evaluation.

**Figure 1:**
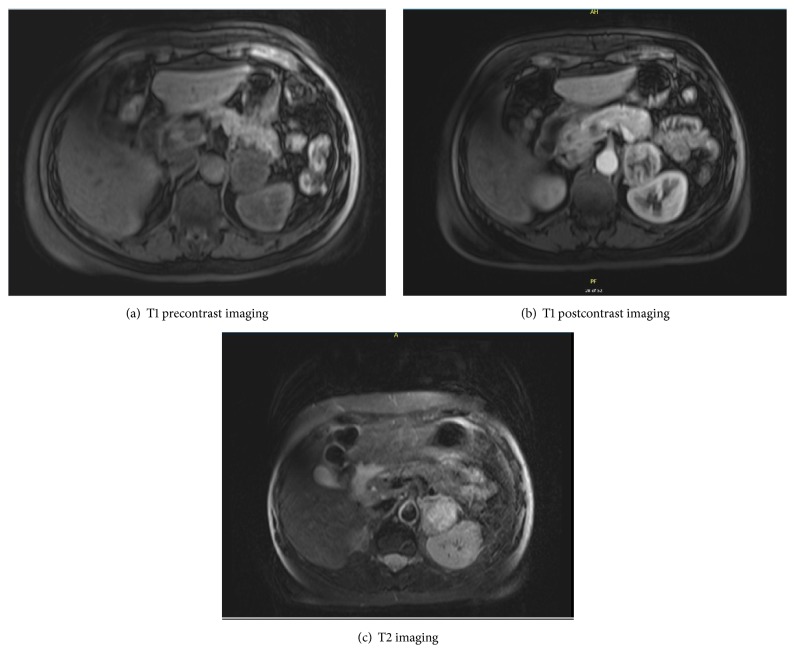
**MRI abdomen with and without contrast. **A 3.4 x 4.0 x 4.7 cm mass is seen in the left adrenal gland. The lesion demonstrated heterogeneous T2 hyperintense/T1 isointense to muscle signal. The T1 signal of the lesion was hypointense relative to the contralateral right adrenal gland.

**Figure 2:**
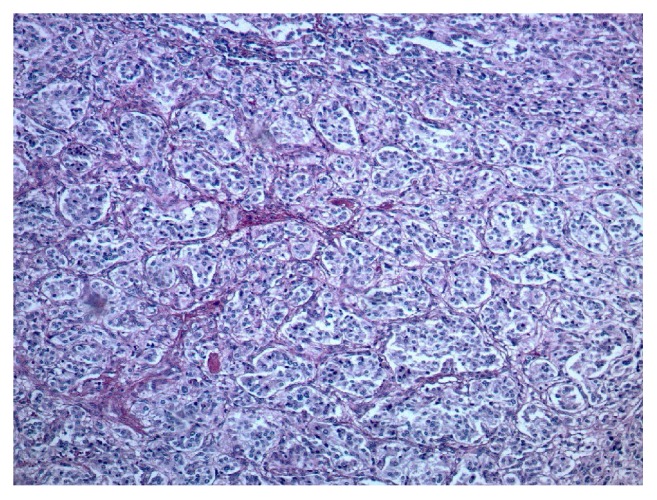
**Pathology from left adrenal.** H&E (100x). Classic pattern of small nests (zellballen) made up of polygonal neuroendocrine cells (chief cells). The nests are separated by interspersed small blood vessels and sustentacular cells.

**Figure 3:**
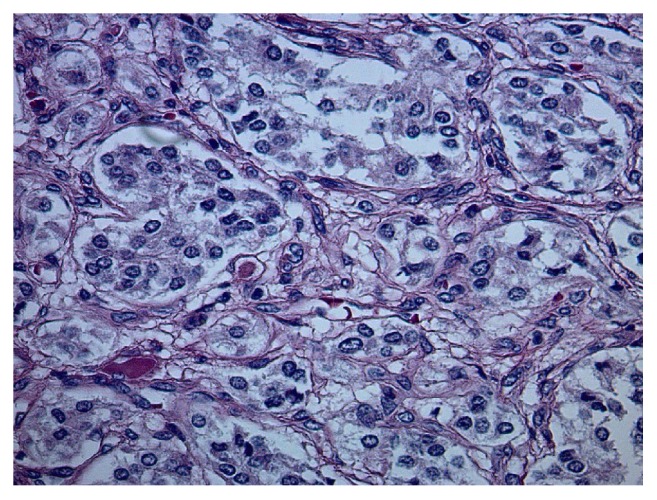
**Pathology from left adrenal.** H&E (400x). High power depicts ample and finely granular eosinophilic cytoplasm. The nuclei exhibit typical “salt and pepper” chromatin, characteristic of all neuroendocrine tumors. Nuclei are round to oval and uniform without significant pleomorphism or mitotic activity. Nucleoli are inconspicuous.

**Figure 4:**
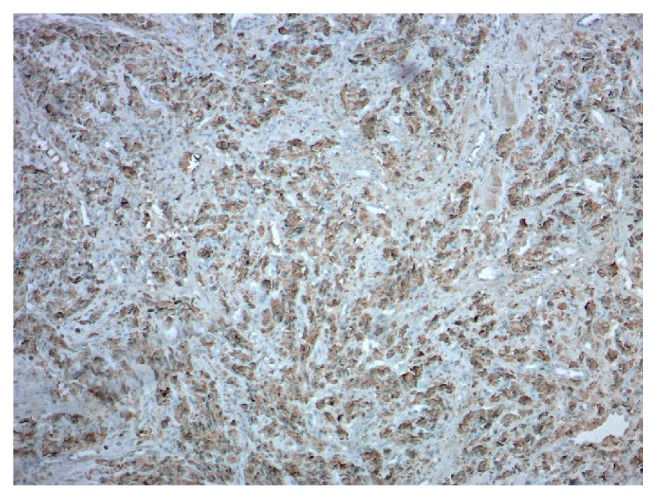
**Pathology from left adrenal.** Chromogranin (100x). Cytoplasmic granular staining diffusely positive in tumor cells.

**Figure 5:**
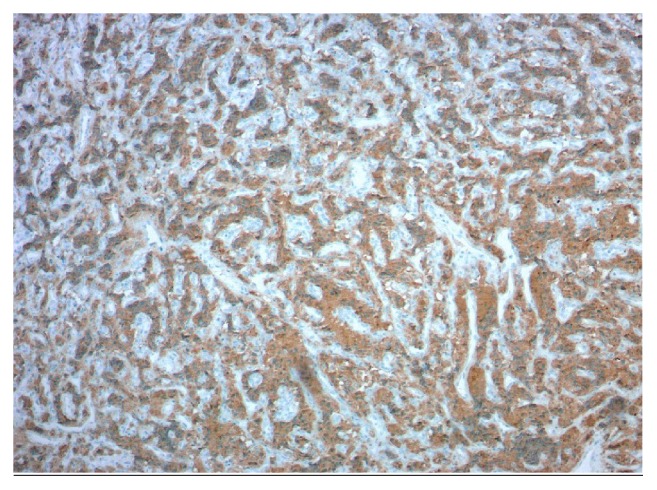
**Pathology from left adrenal. **Synaptophysin (100x). Cytoplasmic granular staining diffusely positive in tumor cells.

She underwent further testing which revealed serum free normetanephrine 344 pg/mL (<=148) and serum free metanephrine <25 pg/mL (<=57). 24-hour urine results included a normetanephrine of 1915 mcg (122-676), metanephrine of 139 mcg (90-315), norepinephrine of 133 mcg/24hr (15-100), dopamine of 148 mcg/24hr (52-480), and creatinine of 893 mg. 24-hour urine free cortisol was normal at 27.4 mcg/24hr (4-50). She was initiated on alpha blockade with phenoxybenzamine and a magnetic resonance imaging (MRI) abdomen was requested. This revealed a 3.4 x 4.0 x 4.7 cm mass in the left adrenal gland which demonstrated heterogenous T2 hyperintense/T1 isointense muscle signal [Figure 1]. She was also evaluated by surgery and underwent a laparoscopic left adrenalectomy in March 2016. Pathology revealed small nests of polygonal neuroendocrine cells which had finely granular eosinophilic cytoplasm, and the nuclei exhibited typical “salt and pepper” chromatin. This was consistent with a pheochromocytoma [Figures 2–5]. Following this she had resolution of all her symptoms and has been afebrile as of 16 months after her surgery. Plasma normetanephrine was 51 pg/mL (<=148), 4 months after surgery, and 24-hour urine normetanephrine was 284 mcg/24hr (122-676), 16 months after surgery.

## 3. Discussion

Pheochromocytomas are rare, but potentially lethal, catecholamine-secreting tumors that arise from the chromaffin cells of the adrenal medulla and sympathetic ganglia. Although their annual incidence is estimated to be less than 1 per 100,000 person-years in the US, this is likely to be an underestimation since a large percentage of patients are only diagnosed at autopsy. It is estimated that 0.5% of all hypertensive patients could have a pheochromocytoma [10]. Pheochromocytomas may occur at any age and sporadic cases are most common in the fourth to fifth decade and are equally common in men and women [3]. These tumors have also been identified as part of familial disorders, such as von Hippel-Lindau (VHL) syndrome, multiple endocrine neoplasia type 2 (MEN2), and neurofibromatosis type 1 (NF1). They are often diagnosed during screening of family members of index cases of these syndromes.

Pheochromocytomas demonstrate a myriad of clinical presentations and this clinical heterogeneity often contributes to delay in diagnosis or even a misdiagnosis. Since almost 90% of all pheochromocytomas are benign and curable when detected early, multiple reports have stressed the need for increased awareness of their heterogenous presentations [10]. While most of the clinical manifestations are thought to be due to excessive catecholamine release, multiple studies have postulated that these tumors also secrete a variety of bioactive neuropeptides and hormones such as interleukin-6 (IL-6), serotonin, and kallikrein that could account for their varied clinical presentations.

The classic clinical triad attributed to pheochromocytomas is paroxysmal headaches, sweating, and palpitations. Other signs and symptoms noted are hypertension (sustained or paroxysmal), pallor, anxiety, tremors, dizziness, dyspnea, and weight loss. There have also been reports of unusual clinical features such as cerebral hemorrhage and hypercalcemia [3, 10]. One of the rarer presentations is presentation as fever of unknown origin.

Fever of unknown origin is a clinical entity that was originally coined to describe presentation of temperature more than 38.3°C lasting more than three weeks, with no diagnosis made despite one week of inpatient investigation. Since then the definition has been revised to expedite workup to include 3 outpatient visits or 3 inpatient days [11]. While the most common cause of FUO remains infections, they only account for a third of all FUOs. [12].

There have only been a handful of case reports that describe extensive workup in the febrile patient, eventually diagnosed as having pheochromocytoma. Martin et al [5] described a 57-year-old male who presented with an acute coronary syndrome and decompensated heart failure. The patient also reported a 3-month history of FUO and a right adrenal incidentaloma. Lab work was then found to be consistent with a pheochromocytoma and the patient's cardiac status improved after a right adrenalectomy. K F Ng [6] described the presentation of a 7-year-old child with a fever of unknown origin. Extensive workup showed a 5cm mass in the left adrenal gland which was thought to be a tumor or an abscess. The patient was directly sent for surgical resection as a pheochromocytoma was not part of the differential diagnosis. The pheochromocytoma was discovered on histology and the patient remained afebrile after surgery. Ciacciarelli et al. [7] described a 45-year-old female who presented with a two-month history of fever. Workup revealed a left adrenal mass, however with normal plasma and urine metanephrine. Interestingly, she had an elevated IL-6. The mass was resected and consistent with a pheochromocytoma and the patient's fevers ceased after the adrenalectomy. Jin et al. [8] describe the case of a 66-year-old male with intermittent fevers and nonoliguric acute renal failure. He was noted on imaging to have a mass in the left adrenal gland which was found to be a pheochromocytoma, and he had normalization of symptoms after adrenalectomy. Yarman et al. [9] describe the case of an 18-year-old female presenting with persistent fevers and weight loss, found after extensive workup to have a right adrenal mass, consistent with pheochromocytoma, with normalization of her symptoms after resection. Interestingly, they documented elevated interleukin-6 levels before surgery, which decreased thereafter.

It is interesting to note that amongst the patients discussed above many did not present with the classical triad of headaches, palpitations, and sweating that is seen with pheochromocytomas. Additionally, some of the patients had elevated interleukin-6 levels, which decreased after resection of the pheochromocytoma [Table 1].

 The exact mechanism of fever in patients with pheochromocytomas is thought to be multifactorial. One theory is that the fever is due to an alteration in the hypothalamic set point which then elevates body temperature [13]. Other causes such as tumor necrosis and excess catecholamines that lead to increased metabolism and peripheral vasoconstriction have also been implicated [14]. It has also been hypothesized that Il-6 produced by the pheochromocytoma is causing the fevers, since tumor removal led to decreased IL-6 levels and resolution of symptoms [7, 9, 15].

Studies have also shown that patients with pheochromocytoma and fevers tend to have larger tumor sizes as well as a higher chance of having tumor necrosis. In a case series, it was also noted that up to half of all patients with pheochromocytoma and fever developed a pheochromocytoma multisystem crisis [13]. Pheochromocytoma multisystem crisis was first described in 1988 and is characterized by the tetrad of high grade fever, encephalopathy, severe hyper/hypotension, and multiorgan failure [16]. This condition can be rapidly fatal and has been treated as a surgical emergency by many, with a mortality rate of up to 50% in the absence of surgical resection [17].

To summarize, pheochromocytomas can present with a plethora of clinical signs and symptoms. Our patient's case, in addition to the above review of the literature, stresses the importance of considering pheochromocytoma in the extensive differential and workup of fever of unknown origin, as a delay in diagnosis puts the patient at risk for severe adverse events from these usually benign tumors. It serves as a teaching point to Internists and Infectious disease specialists to not disregard an adrenal incidentaloma during the workup of a FUO and to the Endocrinologist that high grade fevers in pheochromocytomas can be suggestive of large tumors and an elevated risk of pheochromocytoma multisystem crisis.

## Figures and Tables

**Table 1:** Comparison of our case with other reports of pheochromocytoma presenting as FUO.

**Author and** **Reference**	**Age at** **diagnosis**	**Gender**	**Duration of** **fever**	**Clinical symptoms and signs other than fever**	**Initial labs**: **Metanephrines/Catecholamines/** **Interleukin-6 [IL-6]**	**ESR**	**CRP**	**Size of mass**	**Diagnoses and management**
**Martin et al. [5]**	57 years	Male	3 months	Hypertension, tachycardia, nausea, pallor	Free normetanephrine: 4.82 nmol/L [<0.90] & metanephrine 4.03 nmol/L [<0.50]	Elevated	Elevated	4.5 cm right adrenal mass	Right adrenalectomy with clinical improvement

**K F Ng [6]**	7 years	Male	40 days	Absent hypertension, tachycardia	Not done, as pheochromocytoma not suspected		Elevated	5 cm left adrenal mass	Surgical resection consistent with a pheochromocytoma, followed by clinical improvement

**Ciacciarelli et al. [7] **	45 years	Female	2 months	Hypertension & tachycardia Absent palpitations, headache, diaphoresis.	Urine and plasma metanephrines normal. Interleukin-6: 180.28 pg/mL (3-4)	Elevated	Elevated	3.5 cm left adrenal mass	Left adrenalectomy with clinical improvement

**Jin et al. [8]**	66 years	Male		Hypertension, tachycardia, renal failure	24 hr urine vanillylmandelic acid: 14.3 *µ*mol (9.6–49.5)		Elevated	7 cm left adrenal mass	Left adrenalectomy with clinical improvement and improvement in renal function

**Yarman et al. [9]**	18 years	Female		Weight loss, malaise Absent paroxysmal symptoms	Urine normetanephrine: 3612 *µ*g/24 hours (105–354) Interleukin-6: 12.5 pg/mL (<3.0 pg/mLl	Elevated	Elevated	5.5 cm right adrenal mass	Surgical resection, followed with clinical improvement Interleukin-6 normalized a few weeks after resection

**Our patient**	64 years	Female	1 month	Sweating, headaches, hypertension, malaise	24-hour urine normetanephrine 1915 mcg (122-676)	Elevated	Elevated	5 cm left adrenal mass	Left adrenalectomy with clinical improvement
